# Using patient-derived iPSCs to develop humanized mouse models for chronic myelomonocytic leukemia and therapeutic drug identification, including liposomal clodronate

**DOI:** 10.1038/s41598-018-34193-1

**Published:** 2018-10-26

**Authors:** Kazuki Taoka, Shunya Arai, Keisuke Kataoka, Masataka Hosoi, Masashi Miyauchi, Sho Yamazaki, Akira Honda, Wei Aixinjueluo, Takashi Kobayashi, Keiki Kumano, Akihide Yoshimi, Makoto Otsu, Akira Niwa, Tatsutoshi Nakahata, Hiromitsu Nakauchi, Mineo Kurokawa

**Affiliations:** 1 Department of Hematology and Oncology, Graduate School of Medicine, The University of Tokyo, 7-3-1 Hongo, Bunkyo-ku, Tokyo, 113-8655 Japan; 2 CREST, Japan Science and Technology Agency (JST), 5-7 Chiyoda-ku, Tokyo, 102-0076 Japan; 3 Department of Pathology and Tumor Biology, Kyoto University, Kyoto University, 53 Kawahara-cho, Yoshidakonoecho, Sakyo-ku, Kyoto, 606-8315 Japan; 4 Center for iPS Cell Research and Application, Kyoto University, 53 Kawahara-cho, Shogoin, Sakyo-ku, Kyoto, 606-8507 Japan; 5 Department of Ophthalmology, Graduate School of Medicine, The University of Tokyo, Tokyo, Japan; 6 Division of Stem Cell Therapy, Center for Stem Cell Biology and Regenerative Medicine, Institute of Medical Science, The University of Tokyo, 4-6-1 Shiroganedai, Minatoku, Tokyo, 102-8639 Japan; 7 Division of Stem Cell Processing/Stem Cell Bank, Center for Stem Cell Biology and Regenerative Medicine, Institute of Medical Science, The University of Tokyo, 4-6-1 Shiroganedai, Minatoku, Tokyo, 102-8639 Japan

## Abstract

Chronic myelomonocytic leukemia (CMML) is an entity of myelodysplastic syndrome/myeloproliferative neoplasm. Although CMML can be cured with allogeneic stem cell transplantation, its prognosis is generally very poor due to the limited efficacy of chemotherapy and to the patient’s age, which is usually not eligible for transplantation. Comprehensive analysis of CMML pathophysiology and the development of therapeutic agents have been limited partly due to the lack of cell lines in CMML and the limited developments of mouse models. After successfully establishing patient’s derived disease-specific induced pluripotent stem cells (iPSCs) derived from a patient with CMML, we utilized these CMML-iPSCs to achieve hematopoietic re-differentiation *in vitro*, created a humanized CMML mouse model via teratomas, and developed a drug-testing system. The clinical characteristics of CMML were recapitulated following hematopoietic re-differentiation *in vitro* and a humanized CMML mouse model *in vivo*. The drug-testing system using CMML-iPSCs identified a MEK inhibitor, a Ras inhibitor, and liposomal clodronate as potential drugs for treating CMML. Clodronate is a drug commonly used as a bisphosphonate for osteoporosis. In this study, the liposomalization of clodronate enhanced its effectiveness in these assays, suggesting that this variation of clodronate may be adopted as a repositioned drug for CMML therapy.

## Introduction

Induced pluripotent stem cells (iPSC) technologies have been applied increasingly across clinical medicine and medical research over the last decade^1^. iPSCs for the purposes of disease-modeling and regenerative medicine are two applications that have been widely explored. In disease-modeling studies, cells from patients are reprogrammed to create disease-specific iPSCs and to recapitulate disease-associated phenotypes. Such disease-specific iPSCs can be used as a model in studies on various diseases, and these iPSCs provide a platform for investigating pathophysiology as well as a method for drug testing^2^.

Hematological, disease-specific iPSCs are divided into two groups: those derived from patients with hereditary diseases and those derived from patients with non-hereditary diseases, including acute myeloid leukemia (AML) and myelodysplastic syndrome (MDS)^3^. AML and MDS are difficult to reprogram, although recent studies have reported successful establishment of iPSCs specific to AML and MDS. Applications of these AML and MDS iPSCs demonstrated the possibility of producing disease models and analyzing genetic composition, drug sensitivity, and clonal progression^4–7^.

Chronic myelomonocytic leukemia (CMML) is a clonal hematopoietic stem cell disorder characterized by absolute monocytosis in the peripheral blood (PB) and myelodysplastic along with myeloproliferative properties in the bone marrow (BM)^8–10^. The disease primarily affects the elderly population, with a median age of diagnosis between 65 and 75 years. Prognosis is generally very poor due to the limited efficacy of chemotherapy and to the fact that for many elderly patients, allogeneic hematopoietic stem cell transplantation is not an applicable option. Recently, the hypomethylating drugs azacitidine and decitabine were reported as treatment options for CMML. However, the effectiveness of these drugs such as azacitidine and decitabine remains limited; with overall response rates of 30–40% and complete remission rate of only 7–17%^9,10^. Existing treatments for CMML are insufficient, and novel therapies based on the pathogenesis of CMML are necessary to improve prognosis for the disease. Without available CMML cell lines or adequate models, investigating the pathogenesis of CMML and identifying therapeutic drugs have not been possible. However, with a novel approach of using patient-derived CMML-iPSCs, we were able to establish new platforms for disease modeling and drug testing.

Here, we report various achievements utilizing CMML-iPSCs: hematopoietic re-differentiation *in vitro*, a humanized CMML mouse model via teratomas, and a drug testing system. CMML-iPSCs reproduced disease-specific phenotypes both *in vitro* and *in vivo*. Thus, they provided a powerful channel for dissecting the pathology of CMML and identified liposomal clodronate as a potential repositioned drug for CMML therapy.

## Results

### Generation of iPSCs from a CMML patient sample

Primary samples from one CMML patient and two healthy donors were used after obtaining informed consent. A 75-year-old male, without any past medical history, presented himself with a chief complaint of weight loss, and he was referred to our institution for anemia, monocytosis, and identification of blast cells in the peripheral blood. The bone marrow analysis diagnosed him as CMML-1 with proliferative neoplasms, and his chromosomal study revealed a 46 XY, +1, der (1;7) (q10; p10) karyotype. The collected bone marrow samples in the experiment included CD13+, CD14+ monocytes and 4% of CD34+, CD56+ immature cells in his chronic phase (WBC 43,200/μL (Mono 85.0%, Blast 3.0%). These samples were found with *EZH2*, *NRAS*, and *RUNX1* gene mutations (Supplemental Fig. 1(a)). Fourteen months after diagnosis, his disease progressed from the chronic phase to the transformed phase of leukemia and was resistant to treatment with hydroxyurea.

**Figure 1:**
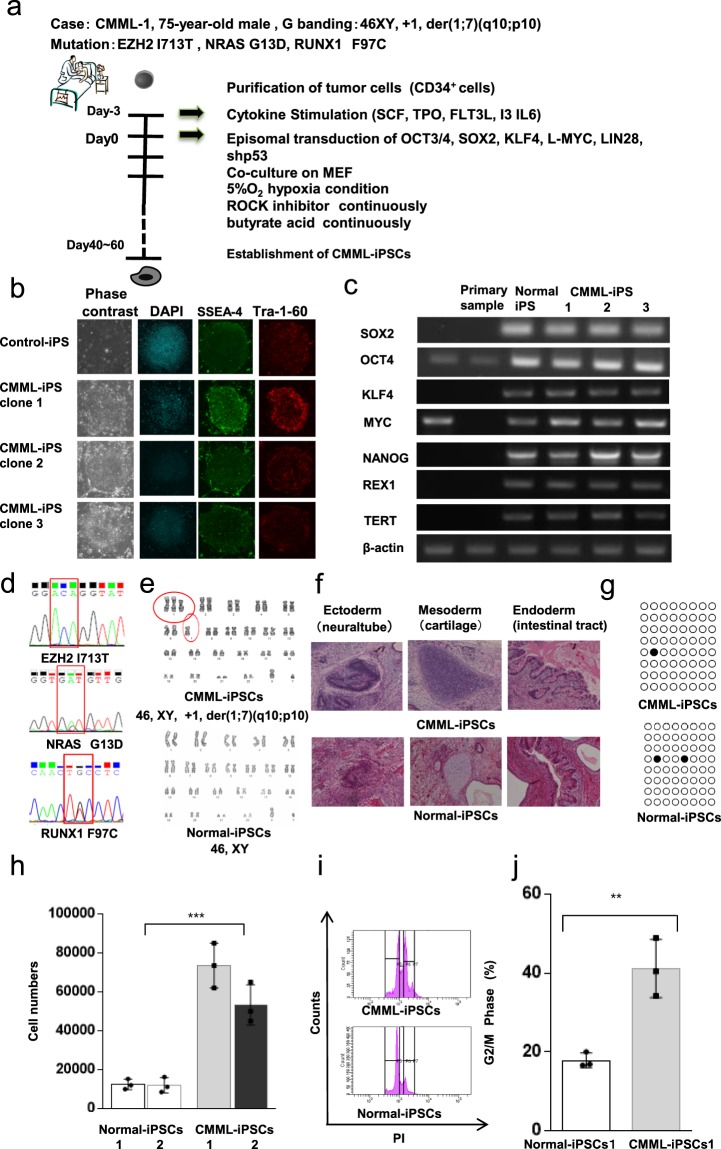
Generation of CMML patient-derived iPSCs. (**a**) Protocol for the generation of CMML patient-derived iPSCs. CD34^+^ cells from patient samples were isolated from BM mononuclear cells. OCT3/4, SOX2, KLF4, L-MYC, LIN 28, and shP53 were transduced using episomal vectors under hypoxic conditions in the presence of a Rho kinase (ROCK) inhibitor and butyrate acid. Three clones of CMML iPSCs from one patient with CMML-1 were established. (**b**) Immunofluorescence staining of pluripotency marker antigens (SSEA-4 and Tra-1-60) in Normal and CMML iPSCs. (**c**) Semi-quantitative RT-PCR of pluripotency markers. The endogenous expression of pluripotent stem cell-specific genes (*OCT3/4*, *SOX2*, *KLF4*, *C-MYC*, *NANOG*, *REX1*, and *TERT*) was confirmed. Each of the images is cropped from different gels. (**d**) *EZH2*, *RUNX1*, and *NRAS* mutations were identified in CMML iPSCs. (**e**) Representative karyotypes of CMML iPSCs showing derivative chromosome (1;7)(q10;p10), an unbalanced translocation, and Normal-iPSCs. (**f**) Histological analyses of teratomas from CMML iPSCs. A teratoma with three germ layers, the ectoderm (neural tube), mesoderm (cartilage), and endoderm (intestinal tract), was observed following H&E staining. (**g**) Bisulfite sequence analysis of the NANOG gene promoter; the black circles represent methylated CpG, while the white circles represent unmethylated CpG. (**h**) CMML iPSCs grew rapidly and displayed a five-fold higher proliferation rate compared to control iPSCs (n = 3 independent experiments, ^***^p < 0.001, CMML-iPSCs 2 clones and control iPSCs 2 clones derived from same donor samples, paired two-sided *t*-test,). (**i**–**j**) Cell cycle analysis showed a relative increase in CMML iPSCs in the G2/M phase. Statistical analysis was performed (n = 3 independent experiments, ^**^p < 0.01, CMML-iPSCs 1 clones and control iPSCs 1 clones derived from same donor samples, Student’s t test). Horizontal lines represent the means ± s.d.

CD34^+^ cells from primary samples of the CMML patient and two healthy control donors were isolated from BM mononuclear cells. OCT3/4, SOX2, KLF4, L-MYC, LIN 28, and shP53 were transduced using episomal vectors under hypoxic conditions in the presence of a Rho kinase (ROCK) inhibitor and butyrate acid^11–13^ (Fig. 1a). Eight clones of CMML-iPSCs from this patient with CMML were established using episomal vectors (Supplemental Table 1). Three stable clones of CMML-iPSCs were obtained. The remaining five clones had differentiation propensity, and they failed to maintain the stable passage cultures of their iPSCs. All three stable clones of CMML-iPSCs were found with 46 XY, +1, der (1;7) (q10; p10), the identical chromosomal abnormality of translocation found in the patient’s original cells. Therefore, these three stable clones of CMML iPSCs were selected for further analyses. Three stable clones of CMML-iPSCs and four Normal-iPSCs clones were obtained from the two healthy donors. CMML-iPSCs displayed the same morphology as that of Normal-iPSCs and expressed pluripotency markers, including SSEA-4 and Tra-1-60 (Fig. 1b and Supplemental Fig. 2a). The endogenous expression of ESC-related transcription factors (OCT3/4, SOX2, KLF4, C-MYC, NANOG, REX1, and TERT) was confirmed by reverse transcriptase PCR (RT-PCR) (Fig. 1c and Supplemental Fig. 2b).

The CMML patient sample and the CMML-iPSCs were screened for genetic mutations in CBL, KRAS, NRAS, IDH1, IDH2, DNMT3A, TET2, ASXL1, RUNX1, JAK2, SRSF2, SF3B1, U2AF35, and EZH2^11^. Both the primary patient sample and all CMML-iPSCs had the same mutations in *EZH2*, *RUNX1*, and *NRAS* (Fig. 1d and Supplemental Fig. 1a). Three Normal-iPSCs were characterized by a 46 XY (Fig. 1e and Supplemental Fig. 1d), and Normal-iPSCs derived from another healthy donor were characterized by a 46XX (Supplemental Fig. 2c). Both CMML and Normal-iPSCs developed into teratomas, containing three germ layers (Fig. 1f and Supplemental Fig. 2d). It has been reported that the cytosine guanine dinucleotides (CpG) in promoter regions, such as *NANOG* and *OCT3/4*, are unmethylated when iPSCs are induced^1^. Based on the study by Takahashi and Yamanaka, we attempted to prove whether the established clones had been reprogrammed as iPSCs (Fig. 1g). In bisulfite genomic sequencing, we revealed that the CpG in the *NANOG* promoter regions of CMML-iPSCs were highly unmethylated, similar to normal iPSCs, implying that the established CMML cells had been successfully reprogrammed as iPSCs^1^.

CMML-iPSCs grew rapidly and displayed a five-fold higher proliferation rate than Normal-iPSCs (Fig. 1h and Supplemental Fig. 2e). The cell cycle analysis revealed a relative increase in CMML-iPSCs in the G2/M phase (Fig. 1i,j).

### The patient’s pathogenesis of CMML was recapitulated *in vitro* in CMML iPSC-derived HPCs

**Figure 2:**
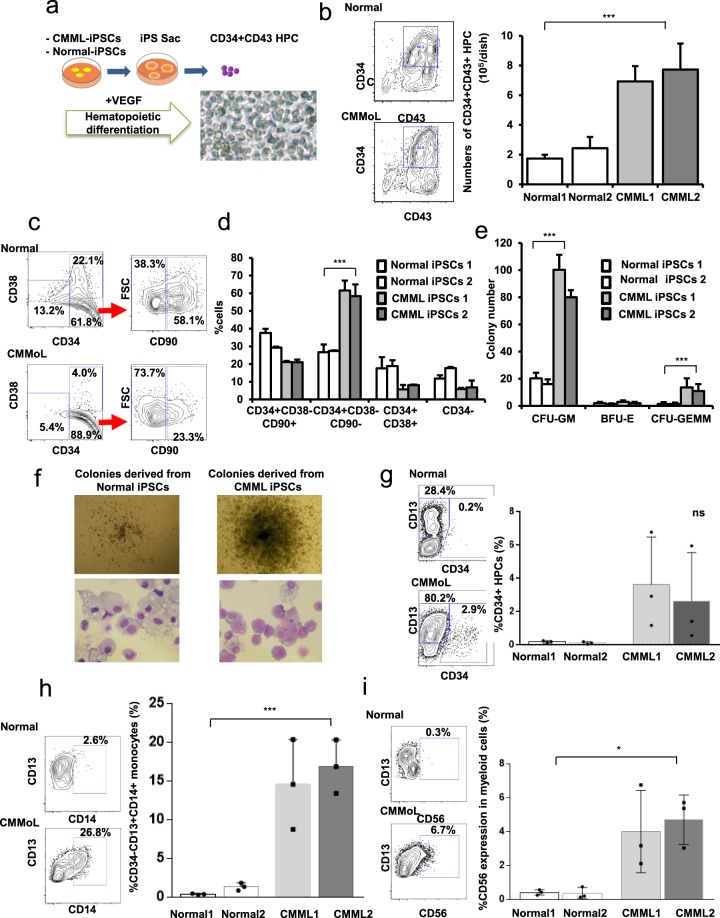
The patient’s pathogenesis of CMML was recapitulated *in vitro* in CMML iPSC-derived hematopoietic progenitor cells (HPCs). (**a**) Scheme for inducing CMML and Normal iPSC-derived HPCs. We obtained CD34 + CD43 + hematopoietic progenitor cells in CMML-iPS-sac on day 17 of the co-culture system. We evaluated two different lines of one normal and unique one CMML iPSC clone in triplicate tests. (**b**) CMML iPSCs generated more CD34^+^ CD43^+^ HPCs than Normal-iPSCs (*n* = 3, independent experiments, ^***^p < 0.001, Normal-iPSCs1 and 2 from a healthy donor, and CMML-iPSCs1 and 2 from a CMML patient, paired two-sided *t*-test). (**c**,**d**) The CD34^+^ CD38^−^ CD90^−^fraction re-differentiated from CMML-iPSCs increased more than Normal-iPSCs (*n* = 3, independent experiments, ^***^p < 0.001, Normal-iPSCs1 and 2 from a healthy donor, and CMML-iPSCs1 and 2 from a CMML patient, Experiments were performed in triplicate. Statistical analyses were performed with ANOVA and the Dunnett post-test for multiple comparisons. ^***^p < 0.001). (**e**) The semi-solid culture of CD34^+^ CD43^+^ HPCs derived from CMML-iPSCs yielded many more CFU-GM and CFU-GEMM colonies, compared to their normal counterparts (*n* = 3, independent experiments, ^***^p < 0.001, Normal-iPSCs1 and 2 from a healthy donor, and CMML-iPSCs1 and 2 from a CMML patient, *Statistical analyses were performed with ANOVA and the* Dunnett post-test for multiple comparisons. ^***^p < 0.001). (**f**) Colonies derived from CMML iPSCs were larger compared to those from Normal-iPSCs. (**g**–**i**) The profiles of the surface antigens in the colonies from CMML iPSC-derived HPCs and controls were analyzed using FACS (*n* = 3, independent experiments, ^*^p < 0.05,^***^p < 0.001, Normal-iPSCs1 and 2 from a healthy donor, and CMML-iPSCs1 and 2 from a CMML patient, paired two-sided *t*-test). Horizontal lines represent the means ± s.d.

Using the previously reported “iPS-sac” method to induce the differentiation of iPSCs into hematopoietic cells^14^, we generated hematopoietic cells from iPSCs. Small clumps, 1 × 10^2^ iPSCs, were transferred to a dish containing irradiated C3H10T1/2 cells. iPSCs with the C3H10T1/2 cells were cultured in differentiation medium with VEGF, which was refreshed every 3 days for 2 weeks. After 2 weeks, these round, hematopoietic-like CD34 + CD43 + HPCs were harvested and sorted by flow cytometry (Fig. 2a). Notably, more CD34^+^CD43^+^HPCs were generated when using CMML-iPSCs as compared to Normal-iPSCs (Fig. 2b and Supplemental Fig. 2f). The CD34^+^ fraction from the primary BM of the CMML patient was merely 2.3%. However, re-differentiated CD34^+^ CD38^−^ CD90^+^ HPCs could be expanded effectively in the differentiated system (Fig. 2c). The CD34^+^ CD38^−^ CD90^−^ fraction re-differentiated from CMML iPSCs increased compared to Normal-iPSCs (Fig. 2d).

To investigate the differentiation capacity, 1 × 10^5^ normal and CMML iPSC-derived CD34^+^ CD43^+^ HPCs were seeded on a semi-solid culture medium. The semi-solid culture of CD34^+^ CD43^+^ HPCs derived from CMML-iPSCs yielded many more large-sized colonies on day 14, which mainly consisted of CFU-GM and CFU-GEMM colonies, compared to their normal counterparts (Fig. 2e). Colonies derived from CMML iPSC-derived HPCs mainly consisted of monoblasts, whereas those from Normal iPSC-derived HPCs mainly consisted of macrophages (Fig. 2f).

Colony assays on each of the three iPSCs clones from one CMML patient and the three clones of Normal-iPSCs from two healthy donors were performed in triplicate (Fig. 2e and Supplemental Fig. 2g). Flow cytometric analysis showed an increase in CD34^+^ HPCs in CMML iPSC-derived hematopoietic colonies (Fig. 2g). Substantial numbers of CD13^+^ CD14^+^ and CD13^+^ CD56^+^ monocytic cells were generated by CMML iPSC-derived HPCs through granulocyte/monocyte terminal differentiation, which recapitulated the representative CD13^+^ and CD14^+^ marker phenotype of CMML (Fig. 2h,i).

### Serial re-plating capacity of hematopoietic progenitors derived from CMML iPSCs

**Figure 3:**
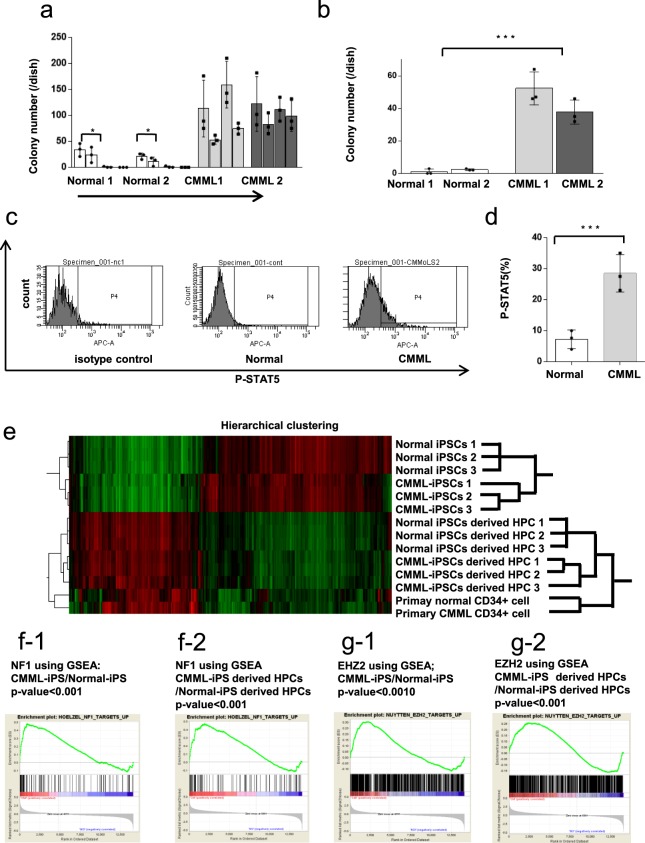
Characteristics of colony formation and genetic analysis of CMML iPSC-derived HPCs. (**a**) CMML iPSC-derived HPCs retained their serial re-plating capacity and generated colonies even after the fourth plating, whereas Normal iPSC-derived HPCs lost the ability to generate colonies after the second plating. CMML iPSC-derived HPCs showed an enhanced self-renewal capacity (*n* = 3, independent experiments, ^*^p < 0.05, Normal-iPSCs1 and 2 from a healthy donor, and CMML-iPSCs1 and 2 from a CMML patient, *Statistical analyses were performed with ANOVA and the* Dunnett post-test for multiple comparisons. ^*^p < 0.05). (**b**) The CMML iPSC-derived HPCs generated hematopoietic colonies under cytokine-deprived conditions. The CMML iPSC-derived HPCs could form hematopoietic colonies, while Normal iPSC-derived cells gave rise to few detectable colonies (*n* = 3, independent experiments, ^***^p < 0.001, Normal-iPSCs1 and 2 from a healthy donor, and CMML-iPSCs1 and 2 from a CMML patient, paired two-sided *t*-test). (**c**) Using pStat5 intracellular flow cytometric analysis, we observed increased phosphorylation of STAT5 in CMML iPSC-derived CD34^+^ CD43^+^ HPCs. (**g**–**i**) The profiles of the surface antigens in the colonies from CMML iPSC-derived HPCs and controls were analyzed using FACS. (**d**) The percentage of pSTAT5 on CMML iPSC-derived CD34^+^ CD43^+^ HPCs was significantly higher than Normal- iPSC-derived HPCs (*n* = 3, independent experiments, ^***^p < 0.001, Normal-iPSCs1 from a healthy donor, and CMML-iPSCs1 from a CMML patient, paired two-sided *t*-test). (**e**) Hierarchical clustering analysis of gene expression patterns. Using hierarchical clustering, these cells were distinguished into two groups: the Normal/CMML iPSC group and Normal/CMML iPSC-derived HPC group. (f-1 and f-2) Expression data were analyzed to identify genes in CMML-iPSC-derived HPCs and CMML-iPSCs that positively affected NF1 using GSEA.(*p* < 0.001). (g-1 and g-2) Expression data were analyzed to identify genes in CMML-iPSC-derived HPCs and CMML-iPSCs that positively affect EZH2 using GSEA. (*p* < 0.001). Horizontal lines represent the means ± s.d.

Subsequently, colony re-plating assays using CMML iPSC-derived HPCs and control iPSC-derived HPCs were performed. CMML iPSC-derived HPCs retained their serial re-plating capacity and generated colonies even after the fourth plating, whereas control iPSC-derived HPCs lost the ability to generate colonies after the second plating (Fig. 3a). Therefore, CMML iPSC-derived HPCs possess the ability of self-renewal. Even when cultured without cytokines, CMML iPSC-derived HPCs formed colonies. In contrast, Normal iPSC-derived HPCs yielded almost no detectable colonies (Fig. 3b). Taken together, these results suggest that CMML iPSC-derived HPCs retained multiple biological properties of CMML.

### Clinical characteristic of increased STAT5 phosphorylation in CMML iPSC-derived HPCs

It has been reported that JMML and CMML cells exhibit hyper-phosphorylation of STAT5 induced by GM-CSF using phospho-STAT5 (pSTAT5) intracellular staining followed by flow cytometric analysis^15^. CD34 + CD43 + iPSCs-derived hematopoietic progenitors from Normal and CMML iPSCs were analyzed for STAT5 hyper-phosphorylation by flow cytometric analysis. Increased STAT5 activation in the CD34 + CD43 + iPSCs-derived hematopoietic progenitors was observed in CMML iPSC-derived HPCs (Fig. 3c,d).

### Up-regulation of NF1 and EZH2 target gene signaling in CMML iPSCs and CMML iPSC-derived HPCs

We performed a hierarchical clustering analysis using clustering software (cluster 3.0). This clustering software can analyze the clustering based on gene expression data. Using this software, the hierarchical clustering separated iPSCs from iPSC-derived HPCs. Furthermore, the categories of CMML-iPSCs were different from those of Normal-iPSCs. Similarly, the categories of CMML-iPSC-derived HPCs were different from Normal-iPSC-derived HPCs (Fig. 3e).

Following, we used gene set enrichment analysis (GSEA) to determine whether specific gene sets correlated with CMML. We performed comprehensive gene analysis to select the gene set that showed significant differences in gene expressions between CMML iPSCs-derived HPCs and normal iPSCs-derived HPCs. GSEA analysis revealed enrichment of many gene sets including NF1 and EZH2 pathways-associated ones in CMML-derived samples. The gene sets commonly enriched in CMML-iPSCs and CMML iPS-derived HPCs we identified are listed in revised supplemental data (Supplementary LIST S1). We focused on NF1 and EZH2 pathways-associated gene sets, because these genes were known to be involved in the pathogenesis of CMML.

CMML-iPSCs were predominantly correlated with the EZH2- and NF1-related genes compared with Normal-iPSCs (Fig. 3f,g-1). Even after the re-differentiation of hematopoietic cells from iPSCs, CMML iPSC-derived HPCs correlated with the EZH2- and NF1-related genes compared with Normal iPSC-derived HPCs (Fig. 3f,g-2).

### Hematopoietic cells generated through *in vivo* teratoma formation from CMML iPSCs

**Figure 4:**
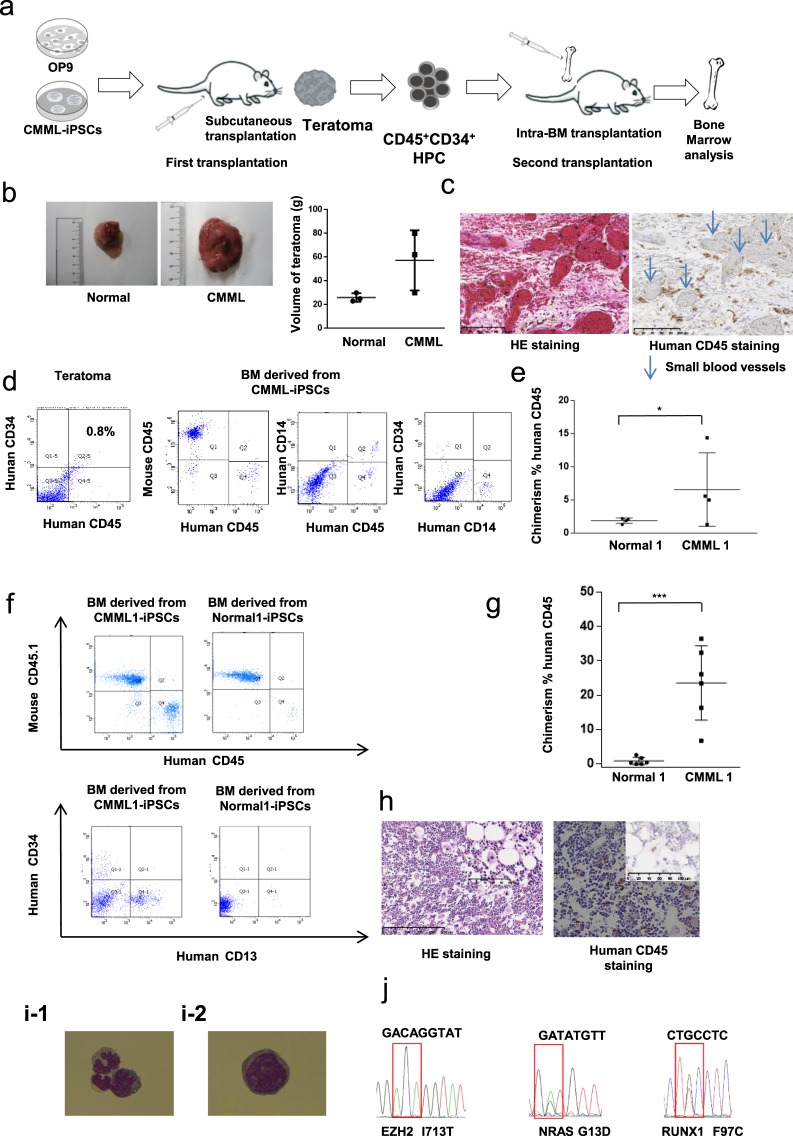
*In vivo* CMML xenograft model recapitulate its pathogenesis. (**a**) Experimental scheme for the first transplantation. We co-injected CMML-iPSCs along with OP9 stromal cells into immunodeficient mice using an *in vivo* system in which human CMML iPSCs develop into HPCs within teratomas. Second transplantation of CMML progenitor cells generated *in vivo*. (**b**) CMML iPS-induced teratomas had larger volumes than the induced teratomas from Normal-iPSCs (*n* = 3, independent experiments, Normal-iPSCs1 from a healthy donor, and CMML-iPSCs 1 from a CMML patient, paired two-sided *t*-test). (**c**) Immunostaining of CD45^+^ blood cells around the small blood vessels generated in the teratoma. (**d**) CD45^+^ CD34^+^ HPCs were isolated from the teratoma. Leakage of the CD45^+^ CMML cells from the teratoma into the BM was observed. (**e**) Estimated number of CMML cells in the BM of engrafted mice in which teratomas formed from tCMML iPS cells (*n* = 4, independent experiments, ^*^p < 0.05, Normal-iPSCs1 from a healthy donor, and CMML-iPSC1 from a CMML patient, paired two-sided *t*-test). (**f**) CMML-iPSC-derived blood *in vivo* reproduced and recapitulated the profiles of CMML surface antigens, CD13+ or CD34+. (**g**) Flow cytometry revealed human chimerism of CD45^+^ cells in the BM of the secondarily transplanted NSG mice. Statistical analysis was performed (*n* = 6, independent experiments, ^*^p < 0.05, Normal-iPSCs1 from a healthy donor, and CMML-iPSCs1 from a CMML patient, paired two-sided *t*-test). (**h**) H&E staining and anti-human CD45 immunostaining. (**i**) Morphological analysis revealed that the sorted CD45^+^ human cells consisted of monocytes and monoblasts (i-1) and a few blasts (i-2). (**j**) *EZH2*, *RUNX1*, and *NRAS* mutations identified in the BM of secondarily transplanted NSG mice. Horizontal lines represent the means ± s.d.

We used the *in vivo* differentiation system to generate transplantable HPCs through teratoma formation^16,17^. A mixture of CMML iPSCs and OP9 cells was injected subcutaneously into 7-week-old NSG mice. Teratomas in the NSG mice were generated 3 months after transplantation (Fig. 4a). The teratomas from CMML iPSCs were larger in volume than those from Normal iPSCs (Fig. 4b). These teratomas contained all three germ layers. Immunohistostaining of human CD45 revealed the presence of human CD45^+^ blood cells, particularly around the small blood vessels in the teratomas (Fig. 4c). Flow cytometric analysis using dissociated teratoma tissues also revealed the presence of human CD45^+^ CD34^+^ HPC fractions. Moreover, in the BM cells of teratoma-bearing mice, human CD45 + CMML cells were detected, which were few CD34 + andCD14 + monocytes (Fig. 4d).

We estimated the number of CMML cells in the BM of the engrafted mice in which teratomas formed from the CMML iPSCs. Human CD45 positive CMML cells were reproduced in the BM and were more abundant than in the normal controls (Fig. 4e).

### Second transplantation of CMML progenitor cells generated *in vivo*

To determine whether these HPCs from CMML iPSC-derived teratomas were transplantable, they were transplanted a second time into the BM of immunodeficient mice (Fig. 4a). Hematopoietic progenitors (HPCs) stained with both human CD45 and human CD34 were isolated from the teratoma and BM cells using a FACS Aria Cell Sorter. Subsequently, 1 × 10^4^ human CD45^+^ CD34^+^ HPCs harvested from the teratoma were injected into the bone marrows of irradiated NSG mice (2 Gy). After 6 months, bone marrow cells from NSG mice were analyzed (Fig. 4a).

CMML-iPSC-derived blood *in vivo* reproduced 23.4 ± 4.421% of the bone marrow and recapitulated the profiles of CMML surface antigens, CD13+ or CD34+. Normal iPSC-derived blood *in vivo* reproduced 0.833 ± 0.437% of the bone marrow in small amounts (Fig. 4f,g). Immunohistochemical staining revealed that human CD45 + cells were engrafted in the BM of recipient mice that received CMML-iPSCs (Fig. 4h). The sorted human CD45^+^ cells consisted of monocytes (Fig. 4i-a) and a few monoblastic cells (Fig. 4i-1,2). Collectively, the CMML marker expression profile was reproduced in this *in vivo* model. *EZH2*, *RUNX1*, and *NRAS* mutations were also identified in the BM of secondarily transplanted NSG mice (Fig. 4j).

### Drug discovery using CMML iPSCs

**Figure 5:**
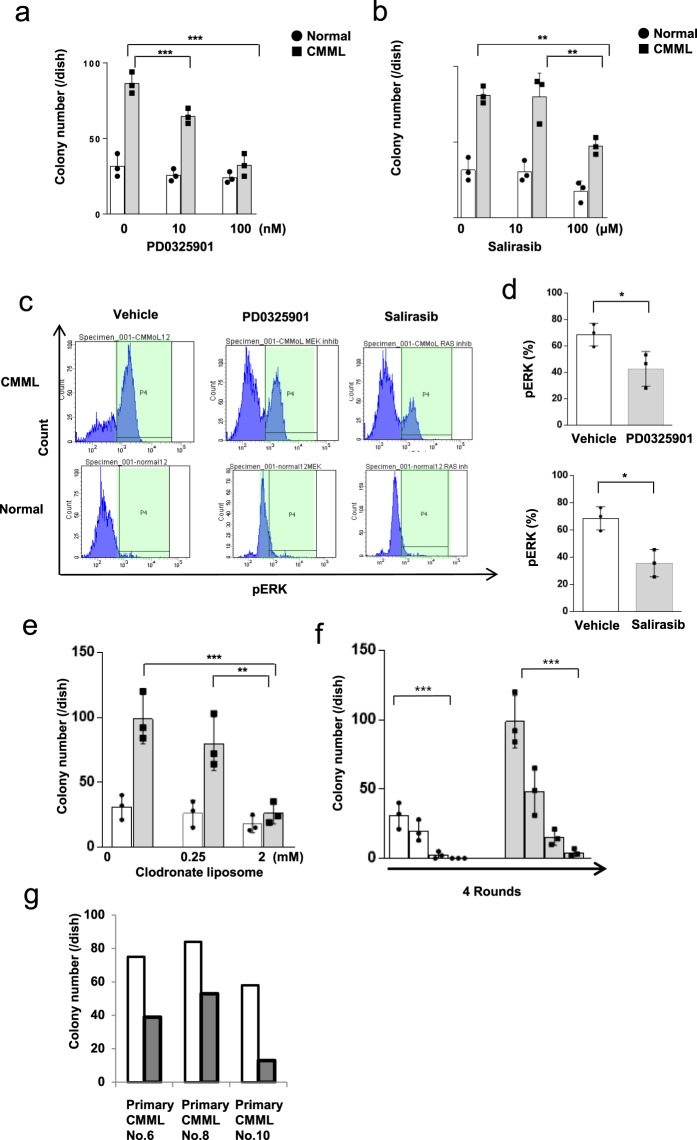
Drug discovery using CMML-iPSCs. (**a**) An MEK inhibitor (PD0325901) reduced the colony-forming capacity of CMML iPSC-derived HPCs (*n* = 3, independent experiments,, Normal-iPSCs1 from a healthy donor, and CMML-iPSCs1 from a CMML patients, Experiments were performed in triplicate. Statistical analyses were performed with ANOVA and the Dunnett post-test for multiple comparisons. ^***^p < 0.001)). (**b**) RAS inhibitor (salirasib) reduced the colony-forming capacity of CMML iPSC-derived HPCs (*n* = 3, independent experiments, Normal-iPSCs1 from a healthy donor, and CMML-iPSCs1 from a CMML patients, Experiments were performed in triplicate. Statistical analyses were performed with ANOVA and the Dunnett post-test for multiple comparisons. ^**^p < 0.01). (**c**) pERK was constitutively activated only in the colonies of CMML iPSC-derived HPCs. Furthermore, ERK phosphorylation was inhibited by MEK and RAS inhibitors in these colonies. (**d**) The expression level of pERK was significantly decreased in CMML-iPSC-derived hematopoietic colonies by MEK or Ras inhibitor (*n* = 3, independent experiments, ^*^p < 0.05, Normal-iPSCs1 from a healthy donor, and CMML-iPSCs1 from a CMML patients, paired two-sided *t*-test). (**e**) Liposomal clodronate suppressed the growth of colonies from CMML iPSCs. Statistical analysis was performed (*n* = 3, independent experiments, Normal-iPSCs1 from a healthy donor, and CMML-iPSCs1 from a CMML patients, Experiments were performed in triplicate. Statistical analyses were performed with ANOVA and the Dunnett post-test for multiple comparisons. ^***^p < 0.001, ^**^p < 0.01,). (**f**) Liposomal clodronate reduced re-plating colonies formed by CMML iPSC-derived HPCs (*n* = 3, independent experiments, Normal-iPSCs1 from a healthy donor, and CMML-iPSCs1 from a CMML patients, Experiments were performed in triplicate. Statistical analyses were performed with ANOVA and the Dunnett post-test for multiple comparisons. ^***^p < 0.001). (**g**) Colony formation by three other primary samples from CMML patients was reduced by liposomal clodronate. Horizontal lines represent the means ± s.d.

We tested the effect of several candidate drugs in order to identify therapeutic agents for CMML. The drug testing method involved three phenotype-screening systems with a colony assay, pERK, and re-plating assay using CMML iPSCs. To evaluate our CMML iPSCs as a tool for cell-based assays, we performed colony assays on CD34^+^ CD43^+^ HPCs differentiated from iPSCs by culturing them with a MEK inhibitor (PD0325901) or Ras inhibitor (salirasib). Treatment with the MEK or Ras inhibitor suppressed colony formation in CMML iPSC-derived HPCs, supporting the utility of these cells for monitoring drug sensitivity in place of primary CMML cells (Fig. 5a,b). ERK, a key downstream effector of Ras, was constitutively phosphorylated only in CMML iPSC-derived HPCs. After treatment with the MEK or Ras inhibitor, the cells from these colonies were harvested on day 14 and analyzed by pERK intracellular phospho-specific flow cytometry, which revealed that ERK phosphorylation was significantly increased in CMML-iPSC-derived colonies compared to Normal-iPSC-derived hematopoietic colonies. ERK phosphorylation was attenuated in CMML iPSC-derived HPCs by the MEK or Ras inhibitor (Fig. 5c). The expression level of pERK was significantly decreased in CMML-iPSC-derived colonies by the MEK or Ras inhibitor (Fig. 5d).

Liposomal clodronate has been reported to deplete monocytes in monocyte/macrophage systems^18^. One of the candidate drugs for CMML, liposomal clodronate, was evaluated in our phenotype-screening system with a colony assay and re-plating assays using CMML-iPSCs. Liposomal clodronate suppressed the growth of colonies from CMML-iPSCs (Fig. 5e). Following, colony re-plating assays using CMML iPSCs and control iPSCs were performed. CMML iPSC-derived HPCs retained their serial re-plating capacity and generated colonies even after the fourth plating (Fig. 3a). However, liposomal clodronate attenuated their serial re-plating capacity and reduced the number of colonies formed by CMML iPSC-derived HPCs (Fig. 5f). Moreover, three other primary CMML bone marrow samples were tested in the colony assay with liposomal clodronate. Colony formation in all CMML patient samples was reduced by liposomal clodronate (Fig. 5g). Clodronate is currently used clinically, and clodronate covered with liposomes represents a potential drug for the treatment of CMML.

## Discussion

Due the lack of cell lines in CMML and the limited developments of mouse models thus far, it has remained difficult to analyze the pathology of CMML and to develop therapeutic agents for the disease. In this study, we developed and utilized CMML-iPSCs to model the disease for purposes of pathophysiological analysis and drug testing to search for potential treatments.

First, we reproduced the hematopoietic system of CMML using CMML-iPSCs. The CMML-iPSCs retained several important disease characteristics of CMML. The blood cells, which were re-differentiated from CMML-iPSCs, recapitulated the pathogenesis of the CMML patient in their myeloproliferation, marker phenotypes, and extensive self-renewal, even after the reprogramming processes. CMML iPSCs-derived HPC from this patient reproduced proliferative monocytosis and demonstrated a serial re-plating ability mimicking a leukemic phase. It has been reported that juvenile myelomonocytic leukemia (JMML) iPSCs exhibit similar characteristics. JMML-derived HPCs demonstrated the proliferative capacities of monocytic cells and activation of the JAK/STAT or MEK/ERK pathway^19,20^. As JMML and CMML may have a common pathophysiology, it is possible that our understanding of these diseases will be advanced by using both CMML-iPSCs and JMML-iPSCs.

Second, we established a humanized CMML mouse model via teratomas using CMML iPSCs *in vivo*. This mouse model makes it possible to analyze the pathogenesis of CMML, explore treatment options for individual patients, and discover other useful insights about the disease’s characteristics. With the development of an *in vivo* transplantation system using teratomas, the formation of vascular niches makes it possible to reconstruct CMML iPSCs-derived from HPCs^16^. Immunohistostaining of human CD45 revealed the presence of human CD45^+^ blood cells, particularly around the small blood vessels in the teratomas. It was recently reported that primary samples of CMML and JMML can be transplanted into NSGS mice that trans-genetically express human GM-CSF, interleukin-3, and stem cell factor to develop remarkably humanized mouse models^21–23^. Our particular technique of developing humanized CMML mouse models using CMML-iPSCs offers an alternative approach that boasts an added advantage: because CMML-iPSCs can be used repeatedly, the mouse models can be produced in infinitely large quantities with limited initial resources.

Furthermore, several drug tests were conducted using CMML-iPSCs, which were established from a CMML patient with genetic mutations in NRAS, RUNX1, and EZH2. Ras and MEK inhibitors effectively suppressed the proliferative capacity of CMML iPSC-derived HPCs. Notably, these drug tests also revealed that liposomal clodronate is a possible agent for treating CMML. Clodronate is a drug used clinically for treatment of osteoporosis, but it has other clinical properties. In particular, clodronate covered with liposomes (liposomal clodronate) can be used to deplete monocytes in monocyte/macrophage systems^18^. In addition, several reports have indicated that injection of liposomal clodronate reduced or depleted monocytes in mice and rats^24–26^. The reduction of monocytes by liposomal clodronate may indicate that liposomal clodronate may be effective in treating CMML and monocytic leukemia. However, this study is the first to attempt administration of liposomal clodronate to the human CMML patient samples. Clodronate has been widely used for the treatment of osteoporosis, and its liposomalization will enable clodronate to be applied to the treatment of CMML as a drug-repositioning treatment.

We acknowledge the limitation of this study in that we obtained and used three clones of CMML iPSCs from only one patient, as it was difficult to reprogram them from CMML patient samples. Our experiment is not the first to suggest that not all samples can be reprogrammed and that samples with chromosome 7 abnormalities are more easily reprogrammed. In the cases of past experiments, samples from MDS patients successfully reprogrammed into iPS cells were MDS samples with t(1;7) derivatives or del(7q), or samples from AML patients with del(7q) complex karyotypes^4^. Past studies have linked successful iPSC reprogramming to the methylation of samples^27^. Additionally, it has been shown that samples with chromosome 7 abnormalities are more easily methylated^28^. Therefore, it may be possible that the methylation of the two t(1;7) derivative samples in our experiment was what enabled their successful reprogramming into iPS cells, whereas our eight other samples, which did not have chromosome 7 abnormalities, could not be reprogrammed.

Therefore, the analysis may not fully reflect all cases of CMML. In order to compensate for this limitation, we utilized patient-derived samples for some parts of this investigation.

## Conclusion

In summary, we established patient-derived CMML-iPSCs and developed various associated systems for disease-modeling and drug-testing purposes: hematopoietic re-differentiation *in vitro*, a humanized CMML mouse models via teratomas, and a drug-screening system using CMML-iPSCs. The clinical characteristics of CMML were recapitulated following hematopoietic re-differentiation *in vitro* and in humanized CMML mouse models *in vivo*. In addition, the well-established drug-testing system indicated the possibility of repositioning liposomal clodronate as a clinically applicable candidate drug for CMML therapy. CMML-iPSCs provide a powerful platform for dissecting the pathology of CMML, and clodronate is a potential repositioned drug for the treatment of CMML.

## Materials and Methods

### Protocol for the generation of patient-derived CMML iPSCs

CD34^+^ cells from patient samples and a healthy control donor were isolated from BM mononuclear cells using FACS Aria. CD34^+^ cells from patient samples were cultured in DMEM, with SCF, TPO, Flat3, IL3, and IL6. DMEM contained 10% FBS, 10 ng/mL IL-3, 10 ng/mL IL-6, and 10 ng/mL SCF. The cultured cells were exposed to episomal vectors expressing five factors (Oct3/4, Sox2, KLF4, L-myc, LIN28, and shTP53). The transfected cells were cultured in a MEF feeder layer. The medium was changed from DMEM to iPS medium on day 0. The iPS medium containing 1 mM butyrate and 10 mM Y-27632 (ROCK inhibitor) was changed every other day. Throughout the process, we used 5% O_2_ hypoxic conditions^12^. After 30 days, ES-like colonies developed in the dish. The colonies were moved to another dish.

### Episomal vector method

This study (approval number 2771) was approved by the ethics committee of the University of Tokyo, and written informed consent was obtained. In addition, all methods in the study were performed in accordance with the relevant stem cell guidelines and regulations established by the University of Tokyo ethics committee. Okita K. and his colleagues have developed efficient methods to generate integration-free human iPSCs^11^. Three episomal plasmid vectors were used: pCXLE-hOCT3/4-shp53-F, pCXLE-hSK (SOX2/KLF4), and pCXLE-hUL (L-MYC/LIN28). The established efficiency of iPS cells can be dramatically enhanced by using an episomal vector encoding EBNA-1 together with three episomal vectors. The episomal vectors disappeared after iPSCs passaged several times. Therefore, analysis of integration-free human iPSCs can be performed without any affects from the transduction of foreign genes in this episomal vector method^11^.

### Mutation analysis of CMML iPSCs

We performed direct genomic sequencing using standard techniques and an ABI 3130 Genetic Analyzer for the patient samples and CMML iPSC samples to screen for the following mutations: *CBL* (exons 8–9); *KRAS* and *NRAS* (exons 1–2); *IDH1* and *IDH2* (exons 4); *DNMT3A* (exons 18–23); *TET2* (exons 3–12); *ASXL1* (exon 12); *RUNX1* (exons 3–8); *JAK2* (V167F); and *SRSF2*, *SF3B1*, *U2AF35*, and *EZH2* (all coding exons)^7,9^.

### Bisulfite sequencing assay

One microgram of genomic DNA was converted using the EpiTect Bisulfite Kit (Qiagen, Hilden, Germany) according to the manufacturer’s recommendations. The NANOG promoter region in the converted DNA was amplified by PCR using previously reported primers. The PCR products were cloned into bacteria using the TA PCR Cloning Kit (DynaExpress, Tokyo, Japan). Ten clones from each sample were analyzed by sequencing with the M13 universal primers^1^.

### “iPS-sac” method to induce iPSCs differentiation into hematopoietic cells

Small clumps (<100 cells) of iPSCs were transferred to a dish containing irradiated C3H10T1/2 cells. iPSCs with the C3H10T1/2 cells were cultured in a differentiation medium containing VEGF, which was refreshed every 3 days for 2 weeks. After 2 weeks, the round, hematopoietic-like cells were harvested and then sorted by flow cytometry^12^.

### Colony forming assay

To investigate differentiation capacity, 1 × 10^5^ control cells and CMML iPSC-derived CD34^+^ CD43^+^ HPC (hematopoietic progenitor cells) were seeded on a semi-solid classical medium (H4434) (Stem cell Technologies) containing recombinant (rh) human stem cell factors, rh GM-CSF, rh IL3, and rh erythropoietin.

### Flow cytometric and morphological analyses

The following antibodies were used for FACS analysis and immunocytochemistry: anti-human CD34 APC-conjugated (Beckman Coulter, Brea, CA, USA), anti-human CD43 PE-conjugated (Beckman Coulter), CD13 APC-conjugated (Beckman Coulter, Brea, CA, USA), CD14 PE-conjugated (Beckman Coulter, Brea, CA, USA), CD24 APC-conjugated (Beckman Coulter, Brea, CA, USA), anti-human CD5 FITC-conjugated (Beckman Coulter), CD7 PE-conjugated (Beckman Coulter), and CD56 PE-conjugated (Beckman Coulter). The cells were analyzed and sorted using a FACS Aria II (Becton Dickinson).

### Microarray analysis

Total RNA was extracted from CD34^+^ cells from a primary CMML patient’s BM and a healthy donor’s BM and their derived iPSCs and HPCs. Samples were analyzed using the Whole Human Genome DNA microarray (Agilent Technologies, Santa Clara, CA, USA). Raw array signals were extracted from the CEL files, and background correction and normalization were performed using RMA. A heat map for selected probes associated with hematopoiesis was produced.

Using the *t*-test, standardized signal values were compared between all pairs of the four groups: normal iPSCs, CMML iPSCs, normal iPSC-derived HPCs, and CMML iPSC-derived HPCs. Any probe sets for which the *p*-values were not <0.05 for any comparisons were excluded from further analysis. Any probe sets for which the maximum/minimum ratio was <4 were also excluded. Using these criteria, 3372 probes were identified. Subsequently, unsupervised hierarchical clustering was performed with Cluster 3.0 (Stanford University), and the results were visualized with Java TreeView. GSEA (www.broadinstitute.org/gsea) was then performed by comparing normal and CMML-derived samples both for iPSCs and HPCs against MSigDB.c2.all.v4.0. Significant gene sets enriched in CMML samples were identified using an FDR q-value < 0.05 and a nominal *p*-value < 0.05.

### Intracellular phospho-specific flow cytometry in phosphorylation of Stat5 and MEK/ERK pathways

CD34 + CD43 + iPSCs-derived hematopoietic progenitors from Normal and CMML iPSCs were analyzed in hyper-phosphorylation of STAT5 by flow cytometric analysis using the Fixation/Methanol Protocol. The cells were fixed with Cytofix/Cytoperm solution (Becton Dickinson; BD) for 20 min at 4 °C and permeabilized with Perm buffer III (BD Perm/Wash™ buffer) for 30 min on ice. The cells were immunostained for pSTAT5 at room temperature for 1 h.

To evaluate our CMML iPSCs as the tools for cell-based assays, we performed colony assays on CD34 + CD43 + HPCs differentiated from iPSCs by culturing them with and without the MEK inhibitor (PD0325901) or the Ras inhibitor (salirasib). After treatment with the MEK or Ras inhibitor, the cells from these colonies were harvested on day 14 and analyzed by pERK intracellular phospho-specific flow cytometry. These cells were intracellularly stained with Phospho-ERK1/2 monoclonal Antibody (APC, eBioscience) using the Fixation/Methanol Protocol. In this protocol, fixation was followed by treatment of cells with methanol. These treated cells were analyzed by pERK intracellular phospho-specific flow cytometry.

### *In vivo* generation of human hematopoietic cells from CMML iPSCs

The study to analyze hematological mouse models using immunodeficient mice (approval number P09-104) was approved by the animal experiment committee of the University of Tokyo. In addition, all methods in the study were performed in accordance with the relevant animal studies guidelines and regulations established by the University of Tokyo ethics committee. A total of 5 × 10^6^ CMML iPSCs and 1 × 10^6^ OP9 cells were injected subcutaneously into the abdomen of 7-week-old NSG mice. Three months after injection, the developed teratomas and BM were harvested. We used four NSG mice for Normal-iPSC-derived teratomas and four mice for CMML-iPSCs via teratomas^14,15^.

### Second transplantation of CMML progenitor cells generated *in vivo*

HPCs stained with both human CD45 and human CD34 were isolated from teratomas and BM cells using a FACS Aria Cell Sorter. Subsequently, 1 × 10^4^ human CD45^+^ CD34^+^ HPCs harvested from the teratoma were injected into the bone marrow of irradiated NSG mice (2 Gy). After six months, bone marrow cells from NSG mice were analyzed^16,17^.

We conducted a transplantable experiment in four mice using normal HPCs from normal-iPSCs-derived teratomas and four mice using CMML HPCs from CMML- iPSCs-derived teratomas.

### Drug discover using patient’s derived CMML iPSCs

We tested the effect of three candidate drugs, a MEK inhibitor (PD0325901), a RAS inhibitor (salirasib), and liposomal clodronate, in order to identify therapeutic agents for CMML. Liposomal clodronate is made of clodronate covered with liposomes. Clodronate has been already approved and is widely used as a bisphosphonate for treating osteoporosis. The drug testing method involved a phenotype screening system with a colony assay, pERK intracellular FACS analysis, and re-plating assay using CMML iPSCs.

### Statistical analysis

Experimental data derived from independent replicates, at least three independent experiments for *in vitro* data, were analyzed using adequate statistics. The significance of the differences between two groups was assessed with a two-sided paired or unpaired *t*-test. Comparisons between more than two groups were performed by ordinary or repeated measures one-way ANOVA analysis of variance with Tukey’s multiple comparisons tests. *p < 0.05, **p < 0.01, ***p < 0.001.

### Study approval

This study was performed in accordance with the Helsinki Declaration and approved by the Research Ethics Board of the University of Tokyo. Written informed consent was obtained from all patients and the healthy donors. All animal experiments were approved by the animal experiment committee of The University of Tokyo and performed according to Animal Care guidelines.

The human genome research ethics committee of The University of Tokyo approves the gene analysis part of this research. This research will be consistent with the guideline of the human genome and gene analysis research.

### Informed consent

Informed consent for experiments involving human samples was obtained from all participants.

## Electronic supplementary material

Supplemental information
