# Supplementary material for: Using patient-derived iPSCs to develop humanized mouse models for chronic myelomonocytic leukemia and therapeutic drug identification, including liposomal clodronate
Source: Sci Rep. 2018 Oct 26;8:15855. doi: 10.1038/s41598-018-34193-1 (PMC6203784; doi:10.1038/s41598-018-34193-1)
Supplement: Supplementary file 1 — Supplemental information [file 41598_2018_34193_MOESM1_ESM.docx]

**Title: Using patient-derived iPSCs to develop humanized mouse models for chronic myelomonocytic leukemia and therapeutic drug identification, including liposomal clodronate**

**Authors:** Kazuki Taoka^1, 2^, Shunya Arai^1, 2^, Keisuke Kataoka^1, 2, 3^, Masataka Hosoi^1, 2^, Masashi Miyauchi^１, 2^, Sho Yamazaki^1, 2^, Akira Honda^1, 2^, Wei Aixinjueluo^5^, Takashi Kobayashi^1, 2^, Keiki Kumano^1, 2^, Akihide Yoshimi^1, 2^, Makoto Otsu^5^, Akira Niwa^4^, Tatsutoshi Nakahata^4^, Hiromitsu Nakauchi^6^, and Mineo Kurokawa^1, 2＊^

**Affiliations**:

^1^Department of Hematology and Oncology, Graduate School of Medicine, The University of Tokyo, 7-3-1 Hongo, Bunkyo-ku, Tokyo 113-8655, Japan

^2^CREST, Japan Science and Technology Agency (JST), 5-7 Chiyoda-ku, Tokyo 102-0076, Japan.

^3^Department of Pathology and Tumor Biology, Kyoto University, Kyoto University, 53 Kawahara-cho, Yoshidakonoecho, Sakyo-ku, Kyoto 606-8315, Japan

^4^Center for iPS Cell Research and Application, Kyoto University, 53 Kawahara-cho, Shogoin, Sakyo-ku, Kyoto 606-8507, Japan

^5^Department of Ophthalmology, Graduate School of Medicine, The University of Tokyo, Tokyo, Japan

^5^Division of Stem Cell Processing/Stem Cell Bank, Center for Stem Cell Biology and Regenerative Medicine, Institute of Medical Science, The University of Tokyo, 4-6-1 Shiroganedai, Minatoku, Tokyo 102-8639, Japan

^6^Division of Stem Cell Therapy, Center for Stem Cell Biology and Regenerative Medicine, Institute of Medical Science, The University of Tokyo, 4-6-1 Shiroganedai, Minatoku, Tokyo 102-8639, Japan

*Corresponding author:

Prof. Mineo Kurokawa, M.D., Ph.D.

Department of Hematology and Oncology, Graduate School of Medicine, The University of Tokyo

7-3-1 Hongo, Bunkyo-ku, Tokyo 113-8655, Japan

E-mail: **kurokawa-tky@umin.ac.jp**

# Supplemental Materials

| **Number** | **Age** | **Disease** | **Chromosome aberration** | **iPSC**  **colony** | **Reprogramming**  **Efficiency** | **Duration** | **Method** |
| --- | --- | --- | --- | --- | --- | --- | --- |
| 1 | 44 | Normal | 46 XY | 36 | 3.6 X 10^-4^ | 20-30 days | 6 factors  Episomal |
| 2 | 75 | CMML-1 | 46 XY, +1der(1;7)(q10:p10) | 8 | 1.2 X 10^-5^ | 40-60 days | 6 factors  Episomal |
| 3 | 74 | CMML-2 | 46XX, +1 der(1;7)(q10:p10)der(20)(q11,2) | 3 | 3 x 10^-5^ | 50-70 days | 6 factors  Episomal |
| 4 | 79 | CMML-2 | 46 XY | 0 | 0 | n.c | 6 factors  Episomal |
| 5 | 66 | CMML-1 | 46 XY,del(13)(q12q14) | 0 | 0 | n.c | 6 factors  Episomal |
| 6 | 80 | CMML-1 | 46 XY | 0 | 0 | n.c | 4 factors  Sendai |
| 7 | 64 | CMML-2 | 46 XX,-7 | 0 | 0 | n.c | 4 factors  Sendai |
| 8 | 75 | CMML-1 | 46 XY, +1der(1;7)(q10:p10) | 0 | 0 | n.c | 4 factors  Sendai |
| 9 | 74 | CMML-2 | 46XX, +1 der(1;7)(q10:p10)der(20)(q11,2) | 0 | 0 | n.c | 4 factors  Sendai |
| 10 | 65 | CMML-2 | 45XY,-7 | 0 | 0 | n.c | 6 factors  Episomal |
| 11 | 35 | Normal | 46 XY | 33 | 3.3 X 10^-4^ | 20-30 days | 4 factors  Sendai |
| 12 | 35 | Normal | 46 XY | 33 | 3.3 X 10^-4^ | 20-30 days | 6 factors  Episomal |
| 13 | 33 | Normal | 46XX | 15 | 1.6 X 10^-4^ | 20-30 days | 6 factors  Episomal |

# Supplementary Tables 1 Reprogramming efficiency of CMML and normal BM cells

6 factor: Non-viral plasmids for generating integration-free human iPSCs encoded OCT3/4, SOX2, KLF4, L-MYC, LIN28, and a short hairpin RNA against TP53.

4 factor; sendai virus: OCT3/4, SOX2, KLF4, c-MYC

**Table S1.** **Reprogramming efficiency of CMML and normal BM cells**

We attempted to establish iPSCs from patients with CMML. However, it was difficult for malignant cells to be reprogrammed using sendai 4 factors. Therefore, we changed the method for reprogramming to establish iPSCs, such as vector, reprogramming factor, additional molecules, and condition. Finally, we used episomal 6 vectors under hypoxic conditions in the presence of a Rho kinase inhibitor and butyrate acid, and we established CMML-iPSCs from two patients. We established iPSCs using BM cells from a CMML-1 patient, a 75-year-old male, which yielded eight stable clones with stable colonies. A CMML-iPSCs from a 74-year-old patient had additional chromosome abnormalities; therefore, we did not select it. A partial reprogramming colony appeared to an embryonic stem cell (ESC)-like colony. However, the partially reprogrammed colony did not produce fluorescence with the Tra-1-60 antibody cells using a live cell imaging method. Efficiency was calculated as the ratio of numbers of CMML-iPSCs to administration numbers of CMML cells.

CMML-1: Myeloblast, monoblast, and promonocyte are < 5% of peripheral blood and < 10% of bone marrow. CMML-2: Myeloblast, monoblast, and promonocyte are 5–19% of peripheral blood and 10–19% of bone marrow. Normal: healthy and patient donors

**Table S2. A list of** **semi-quantitative RT-PCR primers**

**Quantitative RT-PCR**

Total RNA was purified with the NucleoSpin RNA II (Takara-Bio, Shiga, Japan) and used for reverse transcription with the PrimeScript RT Master Mix (Takara-Bio). Primer sequences used to detect endogenous or exogenous expressions of stem cell genes, including Nanog, REL TERT, and endogenous KLF4, SOX2, and OCT4, were as follows. For additional details, see the Supplemental Procedures.

| **Gene** | **Species** | **Forward** | **Reverse** |
| --- | --- | --- | --- |
| hOCT3/4 | Human | GACAGGGGGAGGGGAGGAGCTAGG | CTTCCCTCCAACCAGTTGCCCCAAAC |
| hSOX2 | Human | GGGAAATGGGAGGGGTGCAAAAGAGG | TGCGTGAGTGTGGATGGGATTGGTG |
| hMYC | Human | GCGTCCTGGGAAGGGAGATCCGGAGC | TTGAGGGGCATCGTCGCGGGAGGCTG |
| hKLF4 | Human | ACGATCGTGGCCCCGGAAAAGGACC | TGATTGTAGTGCTTTCTGGCTGGGCTCC |
| hTERT | Human | CCTGCTCAAGCTGACTCGACACCGTG | GGAAAAGCTGGCCCTGGGGTGGAGC |
| REX1 | Human | CAGATCCTAAACAGCTCGCAGAAT | GCGTACGCAAATTAAAGTCCAGA |
| NANOG | Human | CAGCCCCGATTCTTCCACCAGTCCC | CGGAAGATTCCCAGTCGGGTTCACC |

**Supplementary LIST S1. A list of significant gene set in CMML genetics**

We performed comprehensive gene analysis to select the gene set that showed significant differences in gene expressions between CMML iPSCs and normal iPSCs. Moreover we selected the gene set that showed significant differences in gene expressions between CMML iPSCs-derived HPCs and normal iPSCs-derived HPCs below that. We focused on NF1 and EZH2 pathways-associated gene sets, because these genes were known to be involved in the pathogenesis of CMML.

REACTOME_MEIOSIS

REACTOME_MEIOTIC_RECOMBINATION

REACTOME_MEIOTIC_SYNAPSIS

REACTOME_RNA_POL_I_PROMOTER_OPENING

REACTOME_RNA_POL_I_TRANSCRIPTION

REACTOME_TELOMERE_MAINTENANCE

REACTOME_CHROMOSOME_MAINTENANCE

REACTOME_RNA_POL_I_RNA_POL_III_AND_MITOCHONDRIAL_TRANSCRIPTION

KIM_MYCN_AMPLIFICATION_TARGETS_UP

WIERENGA_STAT5A_TARGETS_DN

ACEVEDO_LIVER_CANCER_WITH_H3K27ME3_DN

REACTOME_AMYLOIDS

ACEVEDO_LIVER_CANCER_WITH_H3K9ME3_DN

CAIRO_LIVER_DEVELOPMENT_UP

HOELZEL_NF1_TARGETS_UP

WHITFIELD_CELL_CYCLE_S

ＮＵＹＴＴＥＮ_NIPP1_TARGETS_UP liked to EZH2

OUILLETTE_CLL_13Q14_DELETION_DN

WAMUNYOKOLI_OVARIAN_CANCER_LMP_DN

MOHANKUMAR_TLX1_TARGETS_DN

PLASARI_TGFB1_SIGNALING_VIA_NFIC_1HR_DN

HOEBEKE_LYMPHOID_STEM_CELL_DN

CERVERA_SDHB_TARGETS_2

BENPORATH_OCT4_TARGETS

TAKEDA_TARGETS_OF_NUP98_HOXA9_FUSION_16D_DN

PLASARI_TGFB1_TARGETS_10HR_DNDAVICIONI_PAX_FOXO1_SIGNATURE_IN_ARMS_UP

# Supplemental Figure


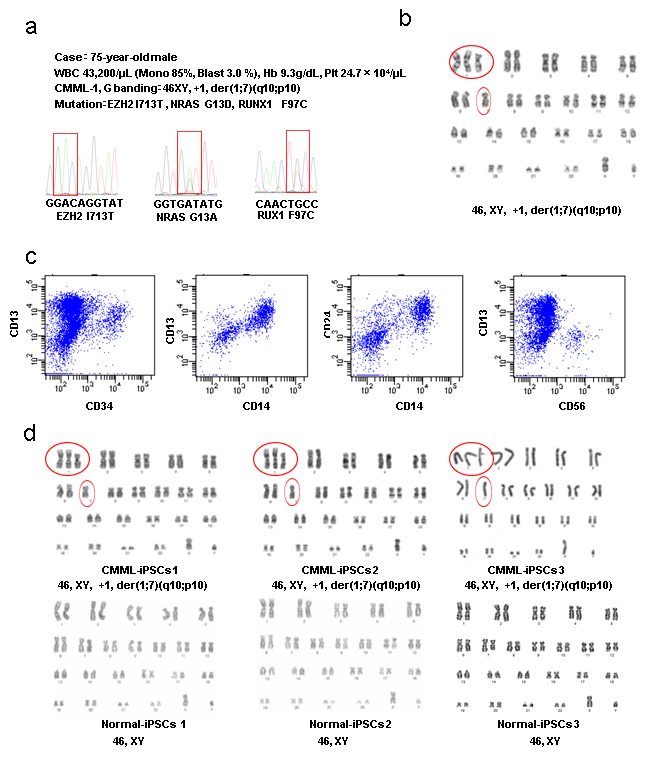


# Supplemental Figure 1.

(a) The case of a 75-year-old male patient with CMML is described (as) with clinical data. A 75 year-old male, without any past medical history, presented himself with a chief complaint of weight loss, and was referred to our institution for anemia, monocytosis and identification of blast cells in peripheral blood. He was found with monocytosis and immature cells (Leucocyte 35,500/μL (Mono 83.0%, Blast 1.5%), Hemoglobin 8.6g/dL, Platelet 11.9×10^4^/ L) in peripheral blood. The bone marrow analysis diagnosed him as CMML-1 with proliferative neoplasms, and his chromosomal study revealed a 46 XY, +1, der (1;7) (q10; p10) karyotype. One year after the initial diagnosis, his bone marrow cells were collected in his chronic phase (WBC 43,200/μL (Mono 85.0%, Blast 3.0%), Hemoglobin 9.3g/dL, Platelet 24.7×10^4^/ L in peripheral blood).

The collected bone marrow samples included CD13+, CD14+ monocytes and 4% of CD34-positive, CD56-positive immature cells. These samples were found with *EZH2*, *NRAS* and *RUNX1* gene mutations (Supplemental Figure 1A). Fourteen months after diagnosis, his disease progressed from the chronic phase to the transformed phase of leukemia (WBC 65,600/μL (Mono 54.5%, Blast 22.0%), Hemoglobin 8.2g/dL, Platelet 13.0×10^4^/ L) and was resistant to the treatment with hydroxyurea. Fifteen months after the initial diagnosis, he died of leukemia.

The patient sample of CMML-1 was screened for genetic mutations, which were identified as *EZH2*, *RUNX1*, and *NRAS*. (b) This patient exhibited the unbalanced chromosomal translocation der(1;7)(q10;p10). (c) Flow cytometric analysis showed an increase in CD34^+^ blasts of primary CMML BM samples. Substantial numbers of CD13^+^ CD14^+^ and CD13^+^ CD56^+^ monocytic cells were recapitulated to have a popular marker phenotype of CMML. (d) Semi-quantitative RT-PCR revealed the exogenous expression of episomal transduced reprogramming factors (OCT3/4, SOX2, KLF4, L-MYC, LIN28, and EBNA) in CMML iPSCs. All episomal-delivered exogenous genes in CMML iPSCs were substantially removed. (e) Representative karyotypes of 3 CMML iPSCs clones showing derivative chromosome (1;7)(q10;p10), an unbalanced translocation, and three normal iPSCs clones.


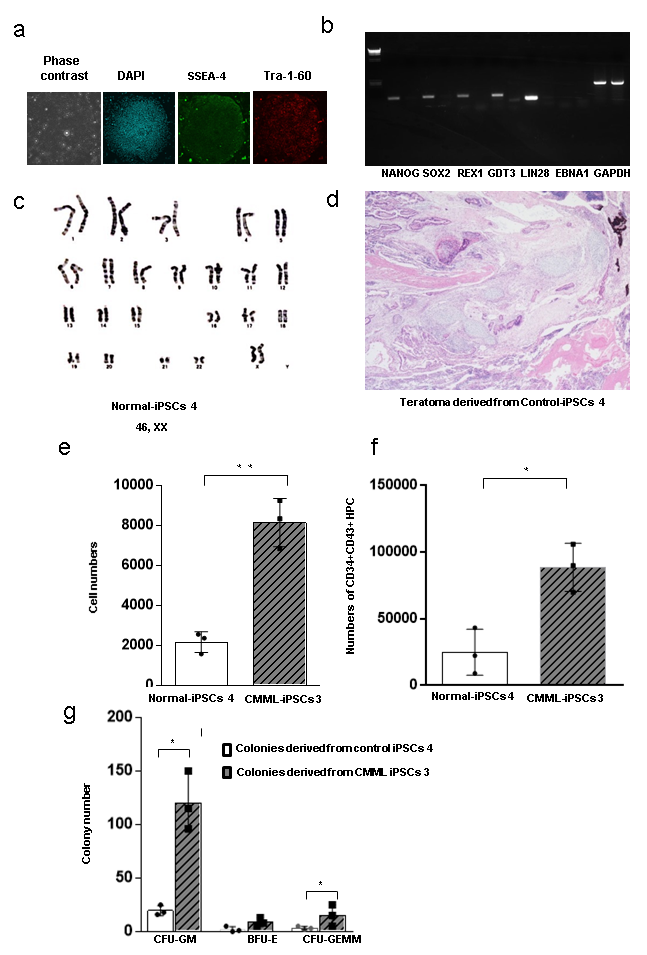


Supplemental Figure 2.　iPSCs clones established from another healthy donor

(a) Immunofluorescence staining of pluripotency marker antigens (SSEA-4 and Tra-1-60) in normal and CMML iPSCs. (b) Semi-quantitative RT-PCR of pluripotency markers. The endogenous expressions of pluripotent stem cell-specific genes were confirmed. (c) Another healthy donor-derived control iPSCs were characterized by 46XX. (d) Histological analyses of the teratoma from CMML iPSCs. (e) CMML iPSCs grew rapidly and displayed a five-fold higher proliferation rate than other control iPSCs (n = 3, independent experiments, ***p* < 0.01, Normal-iPSCs4 from a healthy donor, and CMML-iPSCs3 from a CMML patient, paired two-sided t-test).( f) A larger number of CD34^+^ CD43^+^ HPCs was generated using CMML iPSCs than using other control iPSCs (n = 3, independent experiments, **p* < 0.05, Normal-iPSCs4 from a healthy donor, and CMML-iPSCs3 from a CMML patient, paired two-sided t-test). (g) The semi-solid culture of CD34^+^ CD43^+^ HPCs derived from CMML-iPSCs yielded a much higher number of large-sized colonies than other control Normal-iPSCs from healthy donors (n = 3, independent experiments, ****p* < 0.001, Normal-iPSCs4 from a healthy donor, and CMML-iPSCs3 from a CMML patient, *Statistical analyses were performed with ANOVA and the* Dunnett post-test for multiple comparisons. *p < 0.05).
